# Range Modeling and Surveillance of *Ornithodoros turicata* Ticks: Implications for Detecting African Swine Fever Virus in the United States

**DOI:** 10.1002/ece3.72738

**Published:** 2025-12-19

**Authors:** Christopher J. Butler, Cora P. Garcia, Alexa K. Mendoza, Leila Akhand, Isaac Neuman, Dee B. Ellis, Meriam N. Saleh

**Affiliations:** ^1^ Department of Biology Texas A&M University College Station Texas USA; ^2^ Department of Veterinary Pathobiology, College of Veterinary Medicine & Biomedical Sciences Texas A&M University College Station Texas USA; ^3^ Texas A&M AgriLife Research College Station Texas USA

**Keywords:** ASFv, disease transmission, distribution, ecological niche model, Maxent, *Ornithodoros*

## Abstract

African Swine Fever virus (ASFv) is a re‐emerging global swine disease that, if introduced to the United States, would cause severe economic consequences. The widespread presence of feral hogs in addition to the presence of competent tick vectors, specifically *Ornithodoros turicata* , fosters a greater risk of ASFv establishment. The specific aims of this study were to assess the geographic distribution of *O. turicata* , the spatial distribution of host species richness, and to identify localities that could be monitored for ASFv as part of a comprehensive surveillance system in the United States moving forward. A systematic literature review and field collections were conducted to identify *O. turicata* localities. Ticks were collected from 15/16 surveyed Texas counties and were identified using standard morphological keys and confirmed molecularly on a subset of ticks via amplification and sequencing of a 16S rRNA gene fragment. Ecological niche modeling was used to determine the suite of bioclimatic variables associated with the presence of *O. turicata* based on field collections and the literature review. Six variables of importance were identified: mean temperature of the warmest quarter, mean temperature of the coldest quarter, annual precipitation, precipitation of the wettest month, precipitation of the warmest quarter, and elevation. Subsequently, a map was constructed of the potential distribution of this species, which stretched from southern California to Texas with an allopatric population in Florida. The majority of Texas, with the exclusion of the easternmost quarter of the state, appears to be highly suitable for this species. Host species richness was greatest in Florida and central Texas, suggesting that these regions should be subjected to targeted surveillance priorities. Furthermore, the presence of *O. turicata* in burrows occupied by feral common warthogs ( *Phacochoerus africanus* ) in south Texas is noteworthy, as warthogs are known to sustain ASFv in a sylvatic cycle in Africa. Establishing the current and projected distribution of *O. turicata* is essential to understanding the potential sylvatic cycle of ASFv and creating long‐term surveillance zones if ASFv is introduced to the United States.

## Graphical abstract

*Ornithodoros turicata* collected from a dry ice baited tick trap.

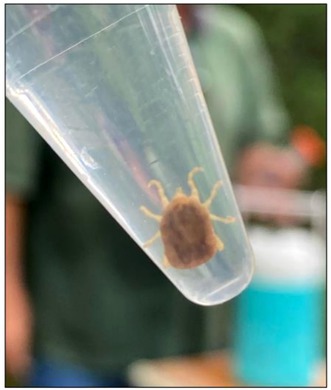

## 1 Introduction

African swine fever virus (ASFv) is a re‐emerging highly contagious and fatal global disease in both domestic and wild swine populations (Sánchez‐Cordón et al. 2018). Endemic to sub‐Saharan Africa, ASFv persists in a sylvatic and domestic cycle among common warthogs ( *Phacochoerus africanus* ), domestic pigs ( *Sus scrofa* ), and argasid ticks in the *Ornithodoros moubata* group (family Argasidae; Penrith et al. 2019). Infected pigs most commonly transmit ASFv to other pigs through direct contact with secretions such as saliva, urine, or manure. Infected swine carry high levels of virus in their blood, and contaminated surfaces (fomites) commonly harbor the virus as well. Wild suids can harbor the virus without showing any symptoms or signs, and warthogs may contract the infection with only a short‐lived viremia, which however can still infect the *Ornithodoros* spp. ticks that may feed on them (Dixon et al. 2019).

Initially restricted to Africa, researchers have now reported ASFv in Europe and Asia including China, the Russian Federation, and Eastern and Central Europe (Wormington et al. 2019). In 2021, Haiti and the Dominican Republic reported ASFv cases, marking the first documentation of ASFv in North America in over 40 years (Onyilagha et al. 2021). Although culling and strict biosecurity measures eventually eradicate most outbreaks, uncontrolled pig movement, spillover events between domestic and feral swine, and the ability of ASFv to persist in uncooked meat products for extended periods of time hinder control efforts (Dee et al. 2018). This can have a substantial economic impact. In Vietnam, for example, an outbreak of ASFv in 2019 resulted in culling approximately 20% of the pig population and a concomitant decline in GDP associated with this outbreak (Nguyen‐Thi et al. 2021). Surveillance efforts have not detected ASFv to date in the United States (US), but the economic impact to the US swine industry would be significant if its presence was ever confirmed (Brown and Bevins 2018).

The growing geographical distribution of ASFv in the Western Hemisphere and the potential severe economic consequences demonstrate the need for additional ASFv surveillance and monitoring in the United States. One avenue of surveillance and monitoring is to identify areas where competent vectors are present. In endemic countries, soft ticks, primarily *Ornithodoros* spp. ticks, serve as vectors and reservoirs for ASFv and infest burrows of wild suids and pens or shelters of domestic suids (Guinat et al. 2016; Sánchez‐Cordón et al. 2018). Transmission of ASFv between wild and domestic pigs occurs when infected warthogs, which serve as asymptomatic carriers, come into close contact with domestic pigs, shedding ASFv through nasal secretions, feces, blood, and urine (Guinat et al. 2016).

*Ornithodoros turicata* serves as a competent vector for ASFv in the lab (Golnar et al. 2019) and feeds on a wide range of vertebrate host species in the United States (Balasubramanian et al. 2024). Given its role as a competent vector, identifying where this soft tick species may occur is essential for evaluating the potential for ASFv spread. Recent work in Florida has documented direct contact between invasive wild pigs and *O. turicata americanus*, reinforcing concerns about the potential for establishment of a sylvatic transmission cycle in this country (Wisely et al. 2025). Previous studies modeled the potential distribution of *O. turicata* tick species in North America (Donaldson et al. 2016), as well as *O. turicata americanus* in Florida (Botero‐Cañola et al. 2024). However, while this model included specimens from the US side of the US–Mexico border, it failed to include any locality data from Mexico resulting in models that show a limited amount of potentially suitable habitat in Mexico, despite multiple records in this country (e.g., Barraza‐Guerrero et al. 2020; Vázquez‐Guerrero et al. 2023).

To address these gaps and build a more comprehensive understanding of ASFv risk in North America, our study expanded upon previous work by incorporating new distribution data and examining ecological factors influencing vector and host presence. The goals of this project were (1) to assess the geographic distribution of *O. turicata* , (2) examine the spatial distribution of host species richness, and (3) identify localities that could be monitored for ASFv as part of a comprehensive surveillance system in the United States moving forward.

## 2 Materials and Methods

### 2.1 Study Sites

Utilizing existing information on the historical occurrence of *Ornithodoros* spp. ticks as well as previously published predictive models (Donaldson et al. 2016; Golnar et al. 2019; Sage et al. 2017; Wormington et al. 2019), we selected potentially suitable tick surveillance sites in suitable habitat in Texas. These sites were in proximity to both large‐ and small‐scale commercial swine operations, and both feral swine and warthog populations in distinct geographic regions of Texas, including sites near the US–Mexico border. We prioritized the selection of locations within or near the USDA APHIS Veterinary Services (VS) Cattle Fever Tick Eradication Program (CFTEP) permanent quarantine zone (PQZ) from Del Rio to Brownsville, in cooperation with USDA VS personnel but also sampled locations north of this zone. We located surveillance sites in a total of 16 counties in Texas (Bexar, Cameron, Carson, Dallam, Dimmitt, Hartley, Hidalgo, Kenedy, Maverick, Randall, Starr, Val Verde, Walker, Webb, Willacy, and Zapata Counties). With permission from the Texas Parks & Wildlife Department (TPWD), we sampled selected wildlife management areas (WMAs) in these counties, as well as privately owned ranches in south Texas with potentially suitable habitat, accessed with landowner approval. We prioritized areas with feral swine and warthog populations as reported by wildlife biologists and private landowners.

We set at least 10 traps in a variety of habitats within each county. *Ornithodoros* spp. ticks inhabit coyote ( *Canis latrans* ) dens, prairie dog ( *Cynomys ludovicianus* ) colonies, rodent (Order Rodentia) and burrowing owl ( *Athene cunicularia* ) nests, in addition to other wildlife dens or burrows (Donaldson et al. 2016). Consequently, we targeted animal burrows and small caves within our selected geographic study sites. Feral swine are habitat generalists (Bevins et al. 2014; Wyckoff et al. 2012), and collared peccary (javelina; *Dicotyles tajacu*) and warthog will also use a wide range of habitats (Green et al. 1985, 2001). Consequently, we also trapped for ticks in locations where these species (e.g., feral swine wallows, warthog burrows, etc.) appeared to spend a substantial amount of time based on visual inspection of ground disturbance and habitat destruction. We recorded latitude and longitude for each sampling site.

### 2.2 Tick Trapping

**FIGURE 1:**
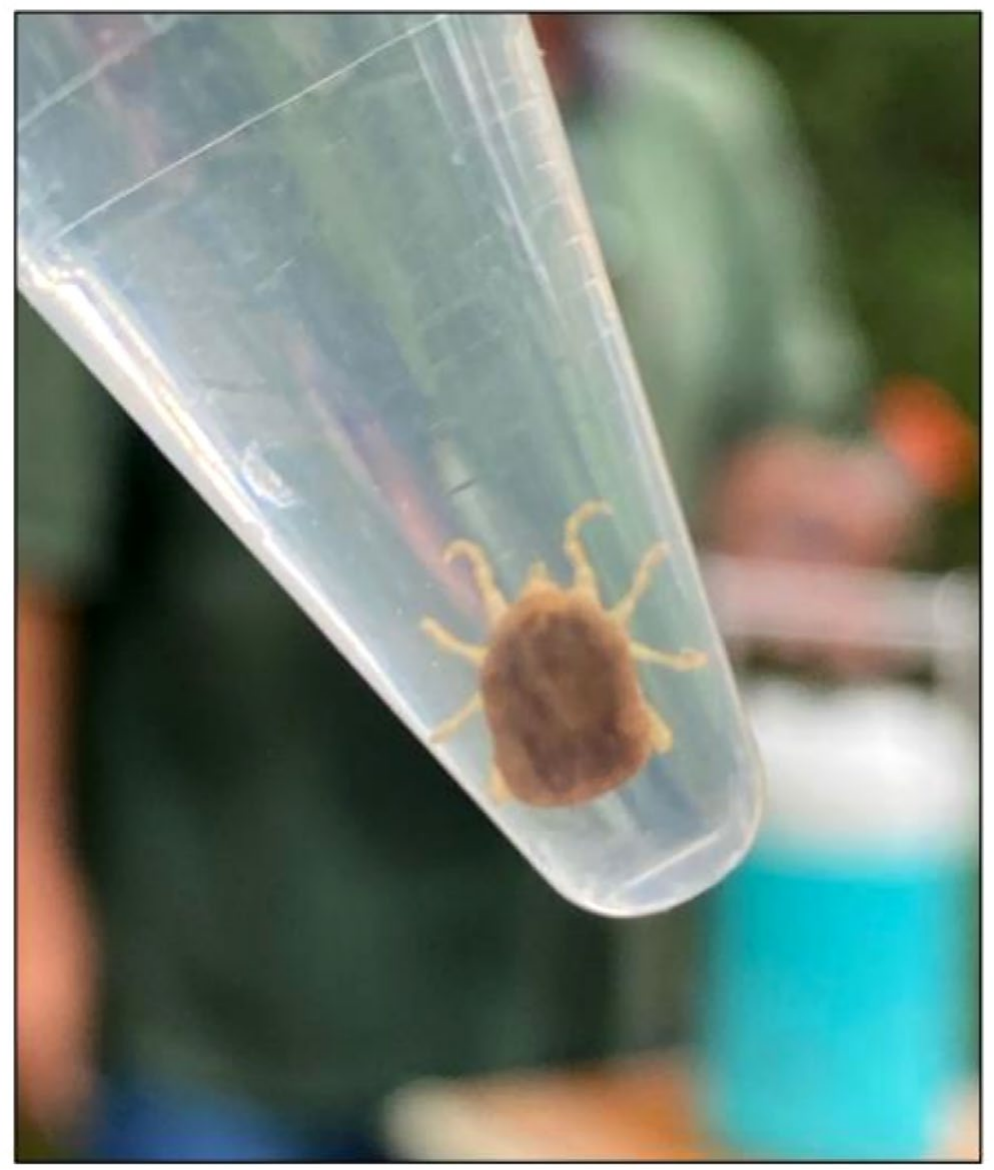
*Ornithodoros turicata* collected from a tick trap.

We used dry ice baited traps to collect *Ornithodoros* spp. ticks (Figure 1). Traps consisted of a 30 cm^2^ piece of cardboard with tape affixed to the outside edges adhesive side up, and with a small cooler mounted in the middle. We baited traps with approximately 125 g of dry ice which sublimate and release CO_2_ attracting host‐seeking ticks. As the ticks moved toward the CO_2_, they became affixed to the adhesive side of the tape. We left dry ice baited traps for a minimum of 12 h and then returned. We removed any ticks affixed to the tape with forceps and placed them in 15 mL conical tubes with 70% ethanol labeled with the location.

We followed the suggestions of Adeyeye and Butler (1991) who recommended that traps should be placed within 4 m of burrows for *Ornithodoros* spp. ticks in Florida. They found that daytime trapping was most successful during May–December, whereas trapping at night was successful all year with the exception of December–January (Adeyeye and Butler 1991). Consequently, we sampled each location during the day and at night to maximize our chances of recovering ticks. *Ornithodoros* spp. tend to avoid wet substrate, so sampling on wet ground did not occur during or within 3 days after heavy rain in that geographic area (Adeyeye and Butler 1991); however, sampling within caves or other protected dry areas still took place as long as the substrate remained dry.

### 2.3 Tick Identification

We examined collected ticks by microscopy to determine the species using standard keys (Cooley and Kohls 1944). Additionally, we recorded the stage and an estimation of “fed” status (i.e., whether the ticks had recently consumed a blood meal). After identification, we preserved specimens in 70% ethanol at −20°C. We dissected a subset of ticks and any damaged specimens that precluded morphologic identification, and we extracted nucleic acid from the midgut and salivary glands for molecular species identification using a 16S rRNA gene fragment (Saleh et al. 2019).

### 2.4 Species Range Modeling

We conducted range modeling for *O. turicata* , following the methods outlined in Phillips et al. (2006) and Phillips et al. (2004). We obtained occurrence data for this species from the Global Biodiversity Information Facility (GBIF.org 2022), a literature review, and field‐collected specimens. Individuals in Florida appear to be a disjunct population (*O. t. americanus*), and although some authors have found that disjunct populations may exhibit minimal differences in responses to bioclimatic variables (e.g., Varaldo et al. 2023), others suggest that modeling disjunct populations does a better job of capturing local adaptation (Chen et al. 2020; Hällfors et al. 2016). Consequently, we modeled the Florida population separately. To process the data and build the models, we followed the procedures described in Butler and Larson (2020). We removed duplicate records and records from outside the native range and resampled the locality data to one record per 25 km^2^. We downloaded elevation and 19 bioclimatic variables from WorldClim (Hijmans et al. 2005) at a resolution of 2.5 arc‐minutes (25 km^2^; Table 1). The spatial extent of the analysis encompassed the continental United States and Mexico.

**TABLE 1:** The ecogeographical variables examined in this study.

Variable	Definition
BIO 1	Annual mean temperature
BIO 2	Mean diurnal range (mean of monthly [max temp − min temp])
BIO 3	Isothermality (BIO 2/BIO 7) × 100
BIO 4	Temperature seasonality (standard deviation × 100)
BIO 5	Max temperature of warmest month
BIO 6	Min temperature of coldest month
BIO 7	Temperature annual range (BIO 5–BIO 6)
BIO 8	Mean temperature of wettest quarter
BIO 9	Mean temperature of driest quarter
BIO 10	Mean temperature of warmest quarter
BIO 11	Mean temperature of coldest quarter
BIO 12	Annual precipitation
BIO 13	Precipitation of wettest month
BIO 14	Precipitation of driest month
BIO 15	Precipitation seasonality (coefficient of variation)
BIO 16	Precipitation of wettest quarter
BIO 17	Precipitation of driest quarter
BIO 18	Precipitation of warmest quarter
BIO 19	Precipitation of coldest quarter
Elevation	Elevation above sea level

We used the regularization approach implemented in ENMtools (Warren et al. 2021) to evaluate models, employing a small sample corrected variant of Akaike's information criterion (AICc) scores (Warren and Seifert 2011). We constructed models using all possible combinations of variables that showed low multicollinearity (i.e., |*r*| < 0.8). We selected variables that contributed the highest predictive information gain and provided unique predictive information. To assess model performance, we calculated area under the curve (AUC) scores using 10‐fold cross‐validation and plotted receiver operating characteristic (ROC) curves by comparing sensitivity to 1 − specificity. We used AICc scores, model weights, and AUC scores collectively to identify the models that best described the current distribution of *O. turicata* . When necessary, we applied model averaging to generate a consensus model. We classified the resulting models into five bands of suitability following the methodology described by Butler et al. (2016): 0%–10% suitable, 10%–20% suitable, 20%–35% suitable, 35%–50% suitable, and > 50% suitable. Finally, we generated response curves for the top model variables to identify the value ranges associated with > 50% suitability.

We obtained shapefiles of host species ranges from GARD (Global Assessment of Reptile Distributions; Caetano et al. 2022; Roll et al. 2017), the IUCN (mammals; IUCN 2024), and BirdLife International and Handbook of the Birds of the World (2022). We gathered county‐level records of feral swine in the United States from USDA‐APHIS (2024) and state‐level records of feral swine from Ortega‐S et al. (2020). Additionally, we also downloaded projected potential feral swine densities from the USDA Forest Service (https://apps.fs.usda.gov/arcx/rest/services/RDW_LandscapeAndWildlife/TCA_Feral_Pig_Density/MapServer), which created the map using models developed by Lewis et al. (2017) and Lewis et al. (2019). Finally, we obtained county‐level records of feral warthogs from Mayer et al. (2020).

## 3 Results

**FIGURE 2:**
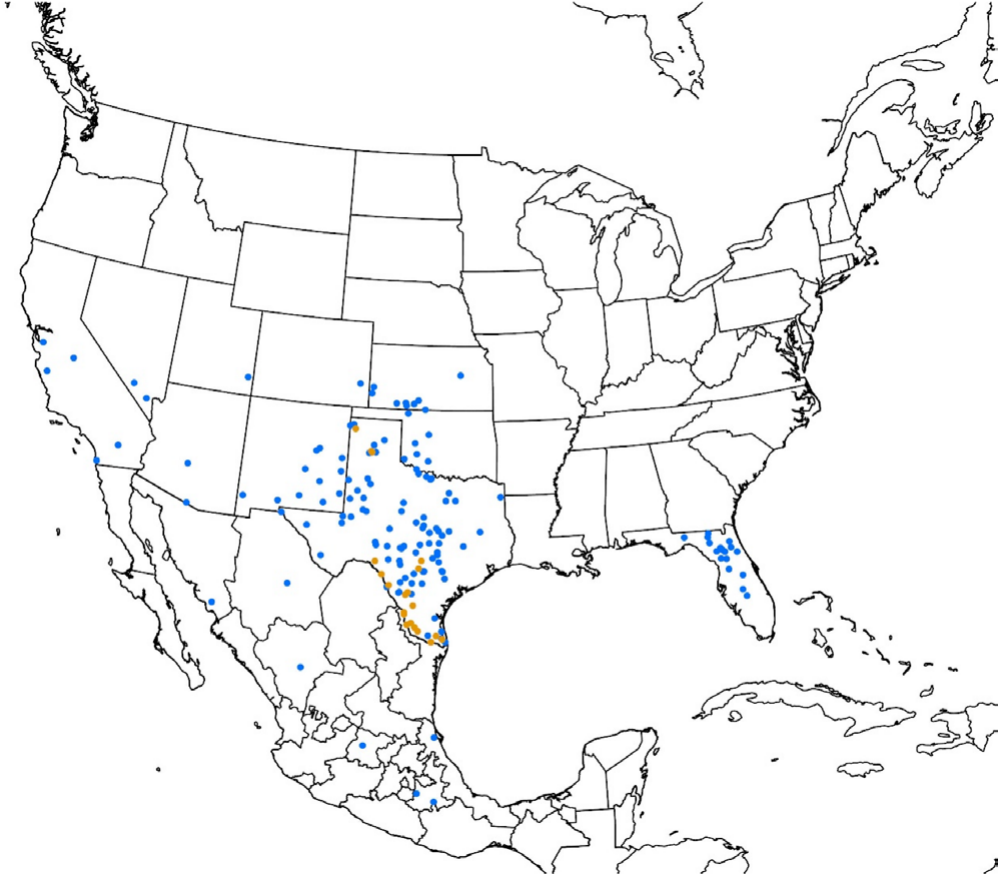
Reported locations (i.e., locations specified in the literature) of *Ornithodoros turicata* specimens in the United States and Mexico are indicated with blue dots, while locations where specimens were collected as part of this study are shown with orange dots.

We collected a total of 1649 *O. turicata* (Figure 1) from Bexar, Cameron, Carson, Dallam, Dimmitt, Hartley, Hidalgo, Kenedy, Maverick, Randall, Starr, Val Verde, Webb, Willacy, and Zapata Counties in Texas (Table 2). The number of ticks trapped in each county ranged from 2 to 987 (median = 28, mean = 109). The majority of collected ticks were nymphs (829/1649), followed by adults (818/1649), with only two larvae recovered. All ticks were collected from wildlife habitat, and adult ticks were recovered from prairie dog holes on premises adjacent to a commercial swine facility in the northern panhandle of Texas. We located *O. turicata* from caves and dens, including warthog dens in Dimmit County and Starr County. Figure 2 shows the combined localities from the literature review, GBIF, and field surveys included in this study, which ranged from central California east to Kansas and south into Mexico, with a disjunct population in Florida.

**TABLE 2:** Counties surveyed for *Ornithodoros turicata* , location/premise type, and number of ticks and stage (A, adult; L, larva; N, nymph) recovered.

County	Location	No. and stage recovered
Bexar	Government Canyon State Natural Area	52 (10N, 42A)
Cameron	Private Ranch	232 (134N, 98A)
Carson	Private Ranch	98 (21N, 77A)
Dallam	Private Ranch	2 (2A)
Dimmit	Chaparral Wildlife Management Area	26 (8N, 18A)
Hartley	Private Ranch	2 (1N, 1A)
Hidalgo	Private Ranch	28 (15N, 13A)
Kenedy	Private Ranch	24 (8N, 16A)
Maverick	Eagle Pass	17 (3N, 14A)
Randall	Palo Duro Canyon State Park	75 (30N, 45A)
Starr	Private Ranch	987 (2L, 543N, 442A)
Webb	Garcia Park	2 (2A)
Willacy	Private Ranch	25 (14N, 11A)
Val Verde	Del Rio	31 (9N, 22A)
Zapata	Private Ranch	48 (33N, 15A)
	Total	1649 (2L, 829N, 818A)

**FIGURE 3:**
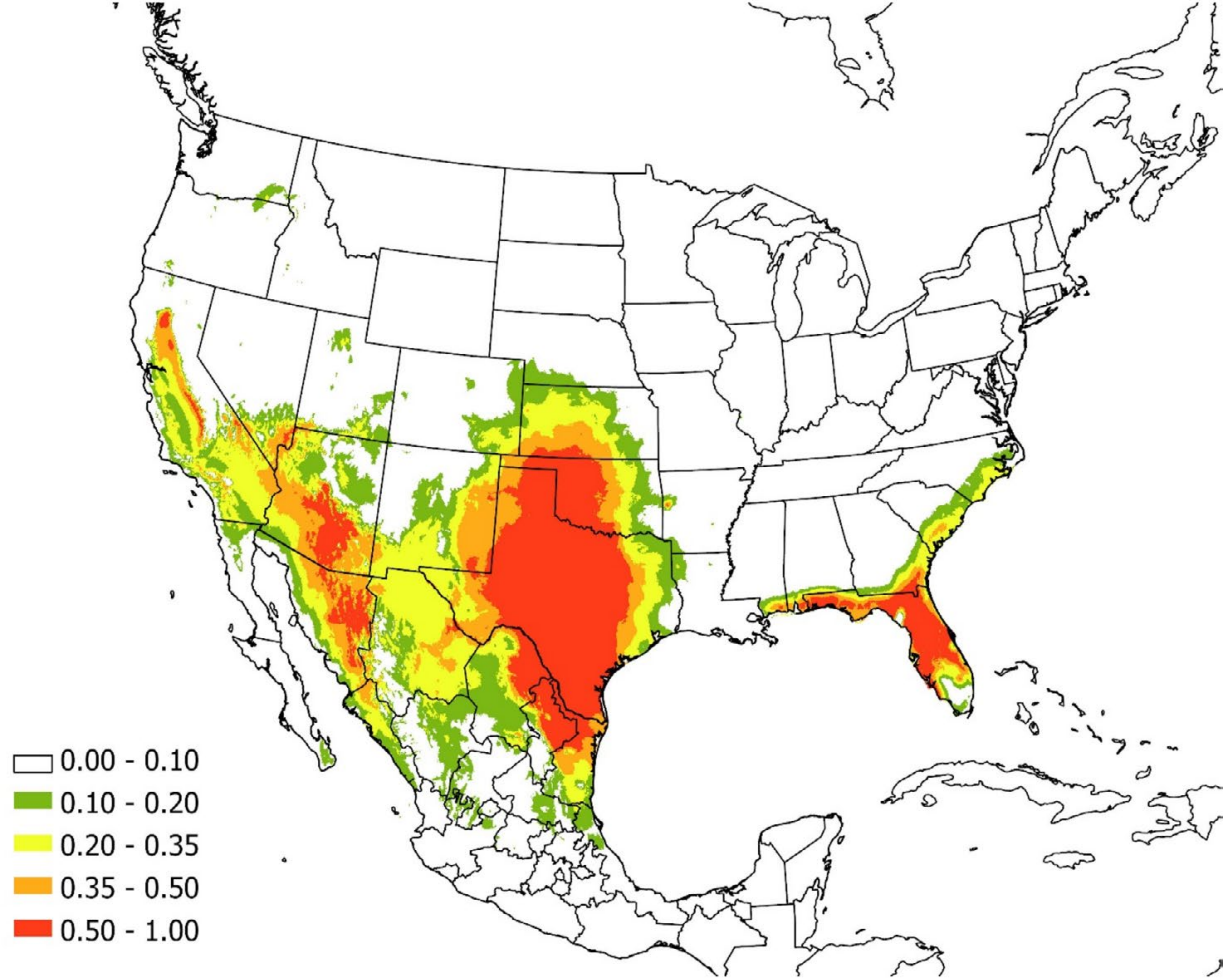
The modeled current distributions for *Ornithodoros turicata* . The legend shows the probability of occurrence, with the red shade representing > 0.5 probability of occurrence.

The best model for the continuous population included *O. turicata* population included mean temperature of the warmest quarter (BIO 10), mean temperature of the coldest quarter (BIO 11), and annual precipitation (BIO 12; Table 3). The AUC for this model was 0.94 ± 0.01. Areas with suitability > 50% had a mean temperature of the warmest quarter of 26.6°C or greater, a mean temperature of the coldest quarter of 4.5°C–14.7°C, and an annual precipitation of 388.9–804.5 mm. Areas of highly suitable bioclimatic conditions extended from extreme southern Kansas (USA) south to Tamaulipas (Mexico), and west to California (USA) and Sonora (Mexico; Figure 3).

**TABLE 3:** The top model runs (i.e., the ones with a ΔAIC_c_ < 2) showing the variables that best describe the distribution of *Ornithodoros turicata* .

Location	Variables	Log likelihood	AIC_c_ score	ΔAIC_c_	wAIC_c_	Mean AUC	*β*
Main range	BIO 10, BIO 11, BIO 12	−1675.28	3420.93	0.00	0.00	0.942 ± 0.008	3.0
Florida	BIO 13, BIO 18	−162.95	333.74	0.00	0.65	0.994 ± 0.004	1.0
	BIO 13, BIO 18, elevation	−159.76	334.98	1.24	0.35	0.996 ± 0.003	1.0

The best model for the disjunct Florida population, however, included precipitation of wettest month (BIO 13), precipitation of wettest quarter (BIO 18), and there was some support for elevation (Table 3). The AUC for the top model was 0.994 ± 0.004. Areas with suitability > 50% had precipitation of the wettest month that ranged from 174.8 to 218.0 mm, precipitation of the wettest quarter that ranged from 489.8 to 632.8 mm, and an elevation that ranged from 18.4 to 51.1 m a.s.l. Areas of highly suitable bioclimatic conditions included most of Florida, excluding extreme southern Florida, and a small area of southeastern Georgia (Figure 3).

**FIGURE 4:**
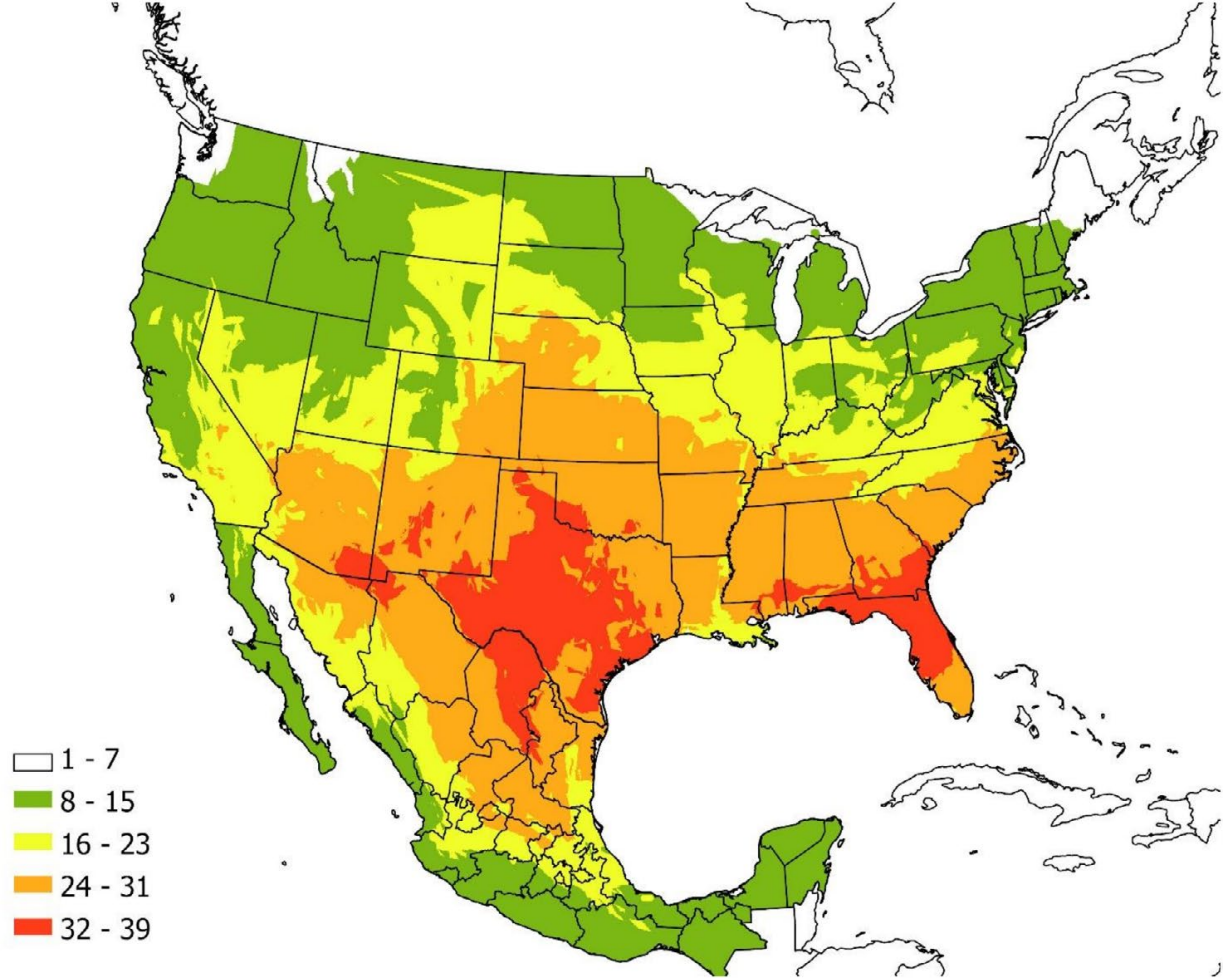
A map overlay showing the number of known and suspected host species. Although 100 species are known or suspected to be hosts for *Ornithodoros turicata* , the maximum number of potential host species in a given location is 39.

We found a total of 100 known and suspected host species in the literature (see Supporting Information). Hosts include a total of five species from class Amphibia, 10 from class Aves, 62 from class Mammalia, and 23 from class Reptilia. Figure 4 shows an overlay of the ranges of the host species. Within the contiguous USA and Mexico, the number of potential host species at any given location ranged from 1 to 39. The greatest diversity of host species occurred from southeastern Arizona east to Oklahoma, Texas, and Nuevo León. Most of Louisiana had lower host species richness, but high numbers of host species extended from extreme eastern Louisiana east through Georgia and extreme southern South Carolina, south to Florida.

**FIGURE 5:**
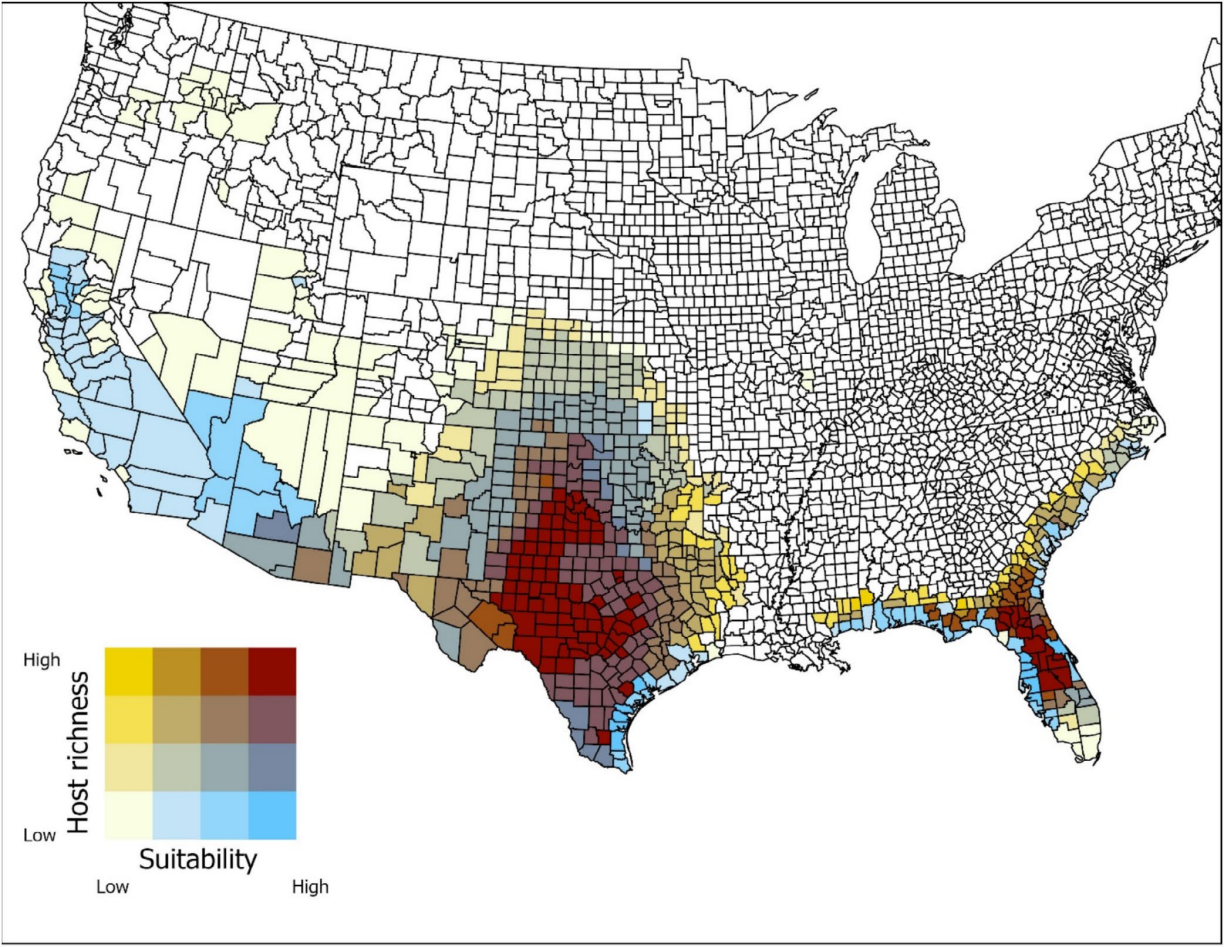
This bivariate choropleth map shows host species richness and projected *Ornithodoros turicata* suitability by county in the United States for those counties where suitability is 10% or greater. Quantiles (10%) were used to assign suitability and species richness. Lighter colors show areas that are less suitable with fewer host species, while darker colors indicate areas of higher suitability and greater numbers of host species.

Figure 5 shows a bivariate choropleth map of suitability and host species richness by county. Locations of high suitability did not overlap perfectly with areas of high host species richness. For example, while parts of California were highly suitable, host species richness was low. Areas that showed the highest suitability for *O. turicata* and relatively high host species richness included the central 1/3 of Texas and peninsular Florida, with areas of relatively high suitability and moderate numbers of host species extending to southeastern Arizona.

**FIGURE 6:**
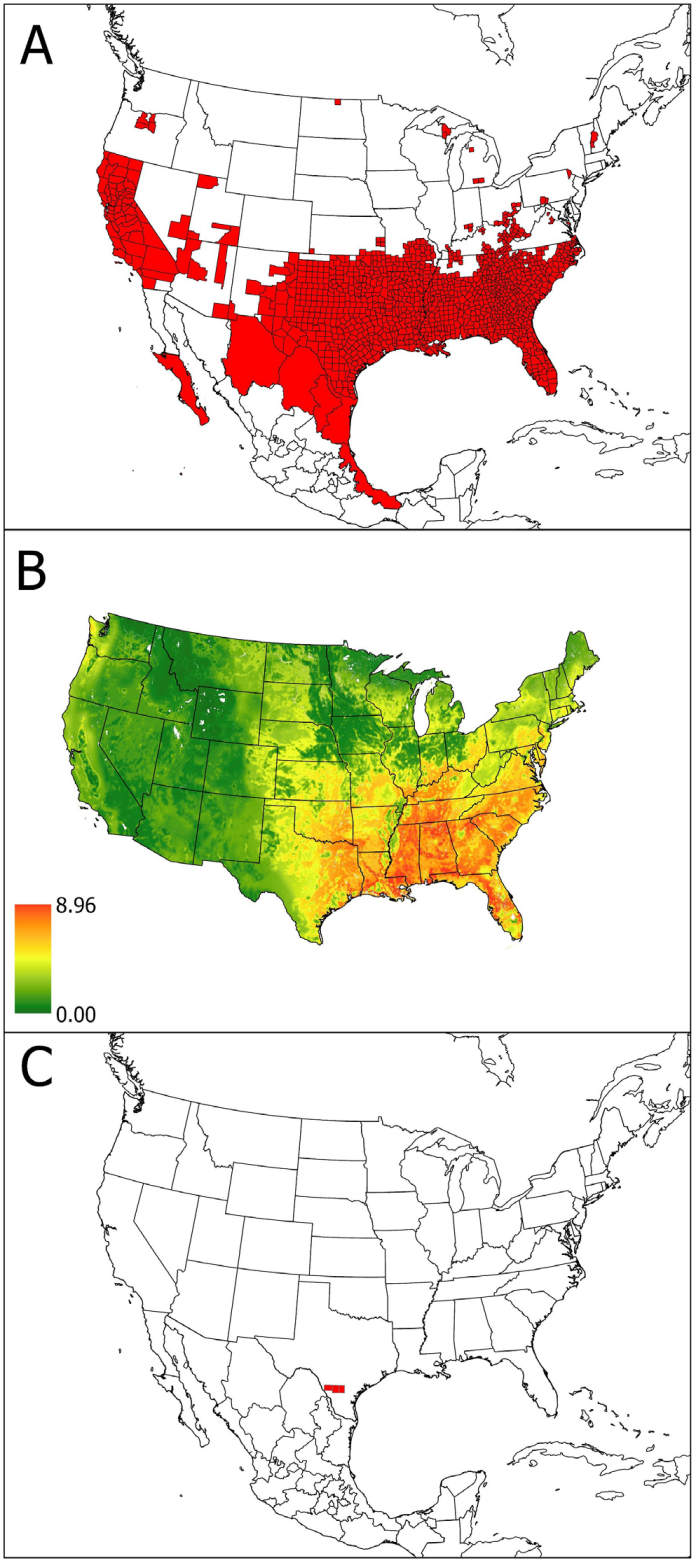
This map shows the range (A) and projected density (B) of feral hogs in the contiguous 48 states, along with the known distribution of the common warthog ( *Phacochoerus africanus* ) in Texas (C).

Figure 6 shows the documented range of feral swine at the state level in Mexico and at the county level in the United States. Feral hogs are present in counties across much of the southern United States (below 37° N), although individuals have been reported from counties as far north as North Dakota (Figure 6A). The greatest modeled density occurs from east Texas north through Oklahoma east to Virginia and south to Florida (Figure 6B). Feral warthogs are present in three counties in southern Texas (Figure 6C), where feral swine density is moderate. This is also in an area where host species richness is relatively poor (Figure 4).

## 4 Discussion

Overall, the modeled distribution appeared to generally match the distribution reported in other studies (Donaldson et al. 2016), although areas of highly suitable habitat were found to extend into Mexico. Although relatively few specimens have been collected from Mexico compared to the United States, multiple studies confirm the species' presence there (Barraza‐Guerrero et al. 2020; Vázquez‐Guerrero et al. 2023), suggesting that the broader distribution predicted by our model is ecologically plausible. The map of the disjunct Florida population also generally seems to agree with the location of collected specimens, although areas of apparently suitable habitat are present in southeastern Georgia where the species has not yet been documented. The predicted distribution is similar to another recent paper (Botero‐Cañola et al. 2024) and agrees that suitable conditions are generally present in Florida north of Lake Okeechobee.

Our top model for the main range identified mean temperature of the warmest quarter (BIO 10), mean temperature of the coldest quarter (BIO 11), and annual precipitation (BIO 12) as the strongest predictors of *O. turicata* occurrence. These variables collectively capture the energetic and hydric constraints that have been identified as being important in determining the distribution of *Ornithodoros* species (Donaldson et al. 2016; Wu et al. 2022): sustained summer warmth (mean temperature of the warmest quarter ≥ 26.6°C) supports development and host‐seeking, while moderate winter temperatures (mean temperature of the coldest quarter ≈4.5°C–14.7°C) limit cold‐related mortality yet avoid prolonged freezing that can suppress activity. Annual precipitation in a semiarid band (annual precipitation ≈389–805 mm) is consistent with this species observed preference for dry, buffered microhabitats (dens, caves, and burrows). Because *O. turicata* is off‐host for extended periods, the macroclimate likely acts through effects on microhabitat humidity and temperature profiles in burrows and caves, and on the availability and persistence of suitable burrow‐forming hosts. In contrast, the disjunct Florida subspecies was best described by precipitation of the wettest month and quarter (BIO 13, BIO 18) and low elevation, suggesting that in Florida, periodic heavy rainfall and low‐relief landscapes more strongly structure microhabitat suitability; ticks may persist in elevated, well‐drained burrow systems (e.g., sandy uplands) despite high ambient moisture.

The number of host species was generally greatest in the areas that were most suitable for *O. turicata* and were particularly large in Florida, Texas, and southeastern Arizona. However, there were some areas, such as California, where *O. turicata* individuals were collected, and habitat suitability was projected to be high (albeit spotty), but host diversity was projected to be low, with only 8–15 host species potentially present. The relatively small number of sites in California with *O. turicata* specimens could be due to the limited number of host species present, as others have suggested that tick abundance is tied to host community composition (Cobbold et al. 2015; O'Neill et al. 2023).

Niche overlap between ticks and hosts is an important component when describing pathogen transmission and reservoirs (Estrada‐Peña and de la Fuente 2016), and clustered associations between hard tick species and host species appear to be due to ecological adaptations and coexistence, rather than to tick‐host coevolution (Estrada‐Peña et al. 2023). Host community composition and density affect tick population dynamics (O'Neill et al. 2023). However, high host richness per se does not necessarily translate to high tick‐host encounter rates for nidicolous soft ticks. Encounter frequencies depend on the density and persistence of burrow systems, the time hosts spend in shelters, and alignment of host activity with tick questing. In Texas, *O. turicata* commonly occupies caves, coyote dens, and suid bedding sites; in Florida, they are frequently associated with sandy upland burrows (e.g., gopher tortoise [ *Gopherus polyphemus* ] burrows) that provide buffered microclimates despite high ambient rainfall. Regions with abundant, well‐drained burrows used repeatedly by suids or mesocarnivores likely yield higher contact rates than areas where hosts are abundant but primarily active in open habitats. Our field detections from warthog dens, including a site that yielded hundreds of ticks over the course of the study, demonstrate that repeated burrow use by suids can support very large local tick populations. For targeted surveillance, this suggests prioritizing burrow‐rich landscapes and microhabitats near suid activity (e.g., wallows, bedding areas, fence‐line crossings, and culverts), particularly in areas with high host species richness. Florida's low elevation, high rainfall systems may concentrate ticks in elevated, xeric sandhill sites, whereas the semiarid settings of the southwestern United States and Mexico provide widespread suitable microhabitats; thus, contact rates may be more spatially clustered for the Floridian subspecies relative to the nominate.

The bivariate choropleth map should be helpful for prioritizing surveillance, as high suitability and high host richness areas may support larger or more stable tick populations and thus pose a greater risk for pathogen transmission. These maps show that the central 1/3 of Texas and peninsular Florida are both areas where there is high suitability and high host species richness. Surveillance efforts in these regions may be especially important for early detection of ASFv or other tick‐borne pathogens, particularly in areas where feral swine and other potential reservoir hosts are also abundant.

The modeled density of feral hogs was greatest in the southeastern United States, and areas of high density overlapped substantially with areas of high suitability for the disjunct population of *O. turicata* in Florida. Modeled density of feral hogs was substantially lower across the main range of highly suitable habitat for *O. turicata* . It should be noted that feral hogs are still in the process of spreading across the United States and Mexico, and so the modeled density does not perfectly align with county records. For example, modeled density is projected to be moderate across much of Kentucky and Virginia but there are currently relatively few county records from these two states (USDA‐APHIS 2024). However, the high density of feral hogs in Florida is a cause for concern as Hess et al. (1987) found that *O. turicata* collected from Florida were capable of becoming infected with ASFv and transmitting to swine. Consequently, if ASFv is introduced into Florida, the high density of feral hogs in this state could potentially spread the virus widely.

Additionally, Texas has been deemed at high risk for introduction of ASFv (Brown and Bevins 2018). Texas has more feral swine than any other state in the United States, with an estimate of 2.6 million (Texas Parks and Wildlife Department 2025). Further, they are found in 253 of the state's 254 counties and thus have a statewide distribution (Texas A&M AgriLife Extension 2025). Because of the risk of introduction from Mexico combined with suitable habitat for the *O. turicata* ticks, and the higher population of feral swine in south Texas, primary surveillance efforts should focus on outdoor swine populations (domestic and feral) in south Texas. Samples will need to be collected from all geographic areas of the state; however, due to the widespread distribution of feral swine and outdoor domestic swine farms, as well as the location of large commercial swine facilities in the Texas Panhandle.

If ASFv is introduced into the United States, the warthogs established in southern Texas could potentially act as a reservoir for this disease as warthogs can be asymptomatic carriers of ASFv (Penrith and Kivaria 2022). We collected *O. turicata* from a culvert that warthogs used in Dimmitt County as well as a warthog den in Starr County. Indeed, the warthog den in Starr County resulted in thousands of individuals of *O. turicata* being sampled over the course of this study. Little has been published about the numbers of warthogs established in southern Texas but it has been suggested that the population is small but growing (Mayer et al. 2020). Warthogs may also be held on private ranches in other parts of Texas, and it is not unheard of for them to escape, so they could potentially act as vectors in other areas of Texas outside of the counties where feral populations are known.

Although *O. turicata* from Florida has been demonstrated to become infected with ASFv and to spread it to pigs, the suitability of *O. turicata* from the main range as a transmission vector has not yet been examined. This knowledge gap is critical, as vector competence can vary geographically due to genetic, environmental, or microbiome‐mediated differences in tick populations. Future work should assess the ability of *O. turicata* from across its range to acquire, maintain, and transmit ASFv. Additionally, it is important to consider that *O. turicata* may play a role in the transmission of other pathogens beyond ASFv. For example, recent work by Mays Maestas et al. (2025) highlights the potential for *O. turicata* to harbor and transmit additional pathogens of veterinary or zoonotic concern such as *Borrelia*. Broader surveillance of pathogen presence in field‐collected ticks, including metagenomic screening, could reveal previously unrecognized disease risks.

In summary, this study demonstrates the value of species distribution modeling for identifying areas at heightened risk for vector‐borne disease transmission. By integrating habitat suitability with host species richness and the distribution of potential reservoirs such as feral hogs and warthogs, we can better prioritize regions for surveillance and management. To build on these findings, future research should assess the vector competence of *O. turicata* populations outside of Florida, survey warthog populations in Texas to clarify their distribution and interactions with native tick species and apply metagenomic tools to detect additional pathogens carried by *O. turicata* . Although our framework was designed to minimize overfitting, residual spatial bias in GBIF and literature‐derived records could still influence model calibration and background sampling. The robust AUC scores for the main range and Florida indicate good discrimination, but future work should incorporate bias files or target‐group background strategies and evaluate sensitivity to additional spatial thinning. Continued addition of systematically collected occurrences from under‐sampled regions in Mexico and the southwestern US will further improve model stability and transferability. Monitoring the continued spread of feral swine into areas of high tick suitability will also be critical, as will coordination among agencies to develop rapid response protocols for early detection of ASFv in both wildlife and ticks. Collectively, these efforts will help mitigate the threat of ASFv and other emerging tick‐borne diseases, particularly in high‐risk regions such as Texas.

## Author Contributions

**Christopher J. Butler:** conceptualization (equal), data curation (equal), formal analysis (equal), investigation (equal), methodology (equal), software (equal), supervision (equal), validation (equal), visualization (equal), writing – original draft (equal), writing – review and editing (equal). **Cora P. Garcia:** investigation (supporting), methodology (supporting), writing – review and editing (supporting). **Alexa K. Mendoza:** investigation (supporting), methodology (supporting), writing – review and editing (supporting). **Leila Akhand:** investigation (supporting), writing – review and editing (supporting). **Isaac Neuman:** investigation (supporting), writing – review and editing (supporting). **Dee B. Ellis:** conceptualization (equal), funding acquisition (equal), investigation (equal), methodology (equal), project administration (equal), resources (equal), supervision (equal), writing – review and editing (equal). **Meriam N. Saleh:** conceptualization (lead), data curation (supporting), formal analysis (equal), funding acquisition (lead), investigation (lead), methodology (equal), project administration (lead), resources (lead), supervision (lead), writing – original draft (lead), writing – review and editing (lead).

## Funding

This work was supported by the US Department of Homeland Security (award no. 18STCBT00001).

## Disclosure

The views and conclusions contained in this document are those of the authors and should not be interpreted as necessarily representing the official policies, either expressed or implied, of the US Department of Homeland Security.

## Conflicts of Interest

The authors declare no conflicts of interest.

## Supporting information


**Appendix S1:** ece372738‐sup‐0001‐AppendixS1.zip.

## Data Availability

Data and code have been uploaded to the Harvard Dataverse and can be accessed at https://doi.org/10.7910/DVN/VQKREO.
