# Supplementary material for: Range Modeling and Surveillance of Ornithodoros turicata Ticks: Implications for Detecting African Swine Fever Virus in the United States
Source: Ecol Evol. 2025 Dec 19;15(12):e72738. doi: 10.1002/ece3.72738 (PMC12717449; doi:10.1002/ece3.72738)
Supplement: Supplementary file 1 — Appendix S1: ece372738‐sup‐0001‐AppendixS1.zip. [file ECE3-15-e72738-s001.zip › ODMAP protocol.docx]

Range modeling and surveillance of Ornithodoros turicata ticks: implications for detecting African Swine Fever virus in the US

– ODMAP Protocol –

Christopher J. Butler, Cora P. Garcia, Alexa K. Mendoza, Leila Akhand, Isaac Neuman, Dee B. Ellis, Meriam N. Saleh

2025-11-24

## Overview

#### Authorship

Contact : [msaleh@cvm.tamu.edu](mailto:msaleh@cvm.tamu.edu)

Study link: N/A

#### Model objective

Model objective: Mapping and interpolation

Target output: Continuous habitat suitability index

#### Focal Taxon

Focal Taxon: Ornithodoros turicata

#### Location

Location: Lower 48 US states and Mexico

#### Scale of Analysis

Spatial extent: -125, -58, 14, 50 (xmin, xmax, ymin, ymax)

Spatial resolution: 2.5 arc-minutes

Temporal extent: N/A

Boundary: political

#### Biodiversity data

Observation type: field survey, citizen science

Response data type: point occurrence

#### Predictors

Predictor types: climatic, topographic

#### Hypotheses

Hypotheses: O. turicata distribution is determined by climatic and topographic constraints

#### Assumptions

Model assumptions: Study species is at equilibrium with the environment. Occurrence data collection is spatially independent

#### Algorithms

Modelling techniques: maxent

Model complexity: Maxent allows for model tuning to balance model complexity and overfitting

Model averaging: AICc (Akaike Information Criterion corrected) was used to determine the best performing model

#### Workflow

Model workflow: We combined point locations obtained with field trapping with locations from the literature and museum data. We then engaged in data cleaning, which included the removal of duplicate records and the resampling of occurrence points to ensure that only one record was retained per 25 km² grid cell. For each unique locality, we extracted associated environmental data, specifically 19 bioclimatic variables and elevation from the WorldClim database. Prior to modeling, we assessed the correlation among predictor variables and retained only those combinations where the absolute value of the pairwise correlation coefficient was less than 0.8, in order to limit multicollinearity. We then constructed species distribution models using Maxent, implemented within ENMtools, and evaluated model performance through 10-fold cross-validation based on the area under the receiver operating characteristic (ROC) curve (AUC). To identify the most informative models, we employed AICc-based model selection and, when appropriate, averaged across the top models to account for model uncertainty. Predicted habitat suitability was classified into categorical bands following Butler et al. (2016). Additionally, we generated response curves for the most important predictor variables to better understand their influence on suitability patterns. Finally, we overlaid the predicted tick distribution maps with shapefiles representing host species ranges, including data obtained from GARD, IUCN, BirdLife International, USDA-APHIS, and other relevant sources.

#### Software

Software: ENMtools, Maxent v3.4.4

Code availability: <https://doi.org/10.7910/DVN/VQKREO>

Data availability: <https://doi.org/10.7910/DVN/VQKREO>

## Data

#### Biodiversity data

Taxon names: Ornithodoros turicata (Dugès, 1876); family Argasidae (soft ticks), order Ixodida; genus Ornithodoros

Taxonomic reference system: n/a

Ecological level: species, populations

Data sources: GBIF.org (21 February 2022) GBIF Occurrence Download <https://doi.org/10.15468/dl.s52nz4>

Sampling design: n/a

Sample size: n/a

Absence data: Absence data for this species has yet to be established

Background data: n/a

#### Data partitioning

Training data: K-fold cross-validation (k = 10) across thinned presence-only occurrences for each dataset (continental US + Mexico; Florida-only). In each fold, 9/10 of presence records used for training; background sampled across accessible area

Validation data: Withheld 1/10 of presence records per fold for validation in 10-fold CV. Model performance assessed via AUC and ROC; model selection by AICc (ENMTools regularization framework).

#### Predictor variables

Predictor variables: Elevation (WorldClim/derived) WorldClim bioclimatic variables BIO1–BIO19 (current climate normals) including BIO1 Annual Mean Temperature BIO2 Mean Diurnal Range BIO3 Isothermality BIO4 Temperature Seasonality BIO5 Max Temperature of Warmest Month BIO6 Min Temperature of Coldest Month BIO7 Temperature Annual Range BIO8 Mean Temperature of Wettest Quarter BIO9 Mean Temperature of Driest Quarter BIO10 Mean Temperature of Warmest Quarter BIO11 Mean Temperature of Coldest Quarter BIO12 Annual Precipitation BIO13 Precipitation of Wettest Month BIO14 Precipitation of Driest Month BIO15 Precipitation Seasonality BIO16 Precipitation of Wettest Quarter BIO17 Precipitation of Driest Quarter BIO18 Precipitation of Warmest Quarter BIO19 Precipitation of Coldest Quarter

Data sources: Hijmans RJ, Cameron SE, Parra JL, Jones PG, Jarvis A (2005) <https://www.worldclim.org>

Spatial extent: -125, -66, 14, 49 (xmin, xmax, ymin, ymax)

Spatial resolution: 2.5 arc-minutes (~0.0417 degrees); cell area ~25 km² in mid-latitudes

Coordinate reference system: WGS84, EPSG:4326

Temporal extent: WorldClim “current” climate normals (baseline period 1970–2000)

#### Transfer data

Data sources: n/a; models were fitted and evaluated under current climate conditions only

<Spatial extent>

Spatial resolution: n/a

Temporal extent: n/a

Models and scenarios: No projections to future climate scenarios; Florida disjunct population modeled separately rather than transferred between regions

Quantification of Novelty: Not performed (no transfers/extrapolations beyond the accessible area); clamping not applicable

## Model

#### Multicollinearity

Multicollinearity: We tested for multicolinearity using Spearman’s rank correlation to account for non-normally distributed data and set a cut off for correlation coefficient r < 0.8 to avoid potential issues with multicollinearity between the variables.

#### Model settings

maxent: featureSet ( candidate feature classes evaluated {L, LQ, LQH}; final selection by AICc), featureRule (select the simplest feature class among models with ΔAICc ≤ 2), regularizationMultiplierSet (candidate values {0.5, 1.0, 1.5, 2.0, 3.0, 3.5., 4.0, 4.5, 5.0}; final selection by AICc), regularizationRule (choose multiplier minimizing AICc; if ΔAICc ≤ 2 across multiples, prefer higher regularization), convergenceThresholdSet (default Maxent convergence threshold 1.0e-5; maximum iterations 500 (defaults)), samplingBiasRule (address sampling bias via spatial thinning of occurrences to one record per 25 km²; random background across accessible area (continental US + Mexico); no bias file or target-group background used), samplingBiasNotes ( GBIF/literature records are clustered; thinning reduces spatial autocorrelation and sampling bias; 10-fold CV helps assess robustness to spatial heterogeneity), targetGroupSampleSize (n/a), offsetSet (n/a), offsetRule (n/a), expertMapProbSet (n/a), expertMapProbRule (n/a), expertMapRateSet (n/a), expertMapRateRule (n/a), expertMapSkewSet (n/a), expertMapSkewRule (n/a), expertMapShiftSet (n/a), expertMapShiftRule (n/a), notes (Florida disjunct population (O. turicata americanus) modeled separately using the same workflow; accessible area (M) defined as the continental US + Mexico; occurrence resampling aligned to WorldClim resolution (2.5 arc-minutes))

Model settings (extrapolation): No projections beyond the accessible area; predictions limited to continental US + Mexico.

#### Model estimates

Coefficients: Maxent produces feature weights (λ) that are not directly interpretable as regression coefficients. We examined model structure via percent contribution and permutation importance per predictor for the selected model(s) and response curves for top variables to identify value ranges associated with >50% suitability

#### Analysis and Correction of non-independence

Spatial autocorrelation: Spatial thinning of occurrences to one record per 25 km² reduces spatial autocorrelation in presences. 10-fold cross-validation evaluates model performance stability across different subsets of thinned presences.

#### Threshold selection

Threshold selection: We did not produce a binary presence/absence map. Instead, we classified continuous suitability into five bands following Butler et al. (2016): 0–10%, 10–20%, 20–35%, 35–50%, and >50% suitability.

## Assessment

#### Performance statistics

Performance on training data: AIC, AUC

Performance on validation data: AIC, AUC

Performance on test data: AIC, AUC

#### Plausibility check

Response shapes: Maxent response curves generated for top-contributing variables in the selected model(s); checked response shapes for ecological plausibility

Expert judgement: map display

## Prediction

#### Prediction output

Prediction unit: logistic for suitability index between 0-1 with 0 being least suitable and 1 being most suitable

#### Uncertainty quantification

Scenario uncertainty: n/a

Novel environments: n/a
